# Supplementary material for: A Critical View of the Application of the APEX Software (Aqueous Photochemistry of Environmentally-Occurring Xenobiotics) to Predict Photoreaction Kinetics in Surface Freshwaters
Source: Molecules. 2019 Dec 18;25(1):9. doi: 10.3390/molecules25010009 (PMC7017383; doi:10.3390/molecules25010009)
Supplement: Supplementary file 1 [file molecules-25-00009-s001.zip › 3.2.4_gcc-4.4.0.zip!/3.2.4_gcc-4.4.0/3.2.4_gcc-4.4.0/doc/octave/html/interpreter/Finding-Elements-and-Checking-Conditions.html]

Finding Elements and Checking Conditions - Untitled


Next: Rearranging Matrices,
Up: Matrix Manipulation


---

### 16.1 Finding Elements and Checking Conditions

The functions `any` and `all` are useful for determining
whether any or all of the elements of a matrix satisfy some condition.
The `find` function is also useful in determining which elements of
a matrix meet a specified condition.

— Built-in Function: **any** (x, dim)  
> For a vector argument, return 1 if any element of the vector is
> nonzero.
>
> For a matrix argument, return a row vector of ones and
> zeros with each element indicating whether any of the elements of the
> corresponding column of the matrix are nonzero. For example,
>
> ```
>           any (eye (2, 4))
>                => [ 1, 1, 0, 0 ]
> ```
>
> If the optional argument dim is supplied, work along dimension
> dim. For example,
>
> ```
>           any (eye (2, 4), 2)
>                => [ 1; 1 ]
> ```

— Built-in Function: **all** (x, dim)  
> The function `all` behaves like the function `any`, except
> that it returns true only if all the elements of a vector, or all the
> elements along dimension dim of a matrix, are nonzero.

Since the comparison operators (see Comparison Ops) return matrices
of ones and zeros, it is easy to test a matrix for many things, not just
whether the elements are nonzero. For example,

```
     all (all (rand (5) < 0.9))
          => 0
```

tests a random 5 by 5 matrix to see if all of its elements are less
than 0.9.

Note that in conditional contexts (like the test clause of `if` and
`while` statements) Octave treats the test as if you had typed
`all (all (condition))`.

— Mapping Function: **xor** (x, y)  
> Return the `exclusive or' of the entries of x and y.
> For boolean expressions x and y,
> `xor (`x`,` y`)` is true if and only if x or y
> is true, but not if both x and y are true.

— Function File: **is\_duplicate\_entry** (x)  
> Return non-zero if any entries in x are duplicates of one
> another.

— Function File: **diff** (x, k, dim)  
> If x is a vector of length n, `diff (`x`)` is the
> vector of first differences
> x(2) - x(1), ..., x(n) - x(n-1).
>
> If x is a matrix, `diff (`x`)` is the matrix of column
> differences along the first non-singleton dimension.
>
> The second argument is optional. If supplied, `diff (`x`,` k`)`, where k is a non-negative integer, returns the
> k-th differences. It is possible that k is larger than
> then first non-singleton dimension of the matrix. In this case,
> `diff` continues to take the differences along the next
> non-singleton dimension.
>
> The dimension along which to take the difference can be explicitly
> stated with the optional variable dim. In this case the
> k-th order differences are calculated along this dimension.
> In the case where k exceeds `size (`x`,` dim`)`
> then an empty matrix is returned.

— Mapping Function: **isinf** (x)  
> Return 1 for elements of x that are infinite and zero
> otherwise. For example,
>
> ```
>           isinf ([13, Inf, NA, NaN])
>                => [ 0, 1, 0, 0 ]
> ```

— Mapping Function: **isnan** (x)  
> Return 1 for elements of x that are NaN values and zero
> otherwise. NA values are also considered NaN values. For example,
>
> ```
>           isnan ([13, Inf, NA, NaN])
>                => [ 0, 0, 1, 1 ]
> ```
>
>
>
>
> **See also:** isna.

— Mapping Function: **finite** (x)  
> Return 1 for elements of x that are finite values and zero
> otherwise. For example,
>
> ```
>           finite ([13, Inf, NA, NaN])
>                => [ 1, 0, 0, 0 ]
> ```

— Loadable Function: **find** (x)  
— Loadable Function: **find** (x, n)  
— Loadable Function: **find** (x, n, direction)  
> Return a vector of indices of nonzero elements of a matrix, as a row if
> x is a row or as a column otherwise. To obtain a single index for
> each matrix element, Octave pretends that the columns of a matrix form one
> long vector (like Fortran arrays are stored). For example,
>
> ```
>           find (eye (2))
>                => [ 1; 4 ]
> ```
>
> If two outputs are requested, `find` returns the row and column
> indices of nonzero elements of a matrix. For example,
>
> ```
>           [i, j] = find (2 * eye (2))
>                => i = [ 1; 2 ]
>                => j = [ 1; 2 ]
> ```
>
> If three outputs are requested, `find` also returns a vector
> containing the nonzero values. For example,
>
> ```
>           [i, j, v] = find (3 * eye (2))
>                => i = [ 1; 2 ]
>                => j = [ 1; 2 ]
>                => v = [ 3; 3 ]
> ```
>
> If two inputs are given, n indicates the maximum number of
> elements to find from the beginning of the matrix or vector.
>
> If three inputs are given, direction should be one of "first" or
> "last", requesting only the first or last n indices, respectively.
> However, the indices are always returned in ascending order.
>
> Note that this function is particularly useful for sparse matrices, as
> it extracts the non-zero elements as vectors, which can then be used to
> create the original matrix. For example,
>
> ```
>           sz = size(a);
>           [i, j, v] = find (a);
>           b = sparse(i, j, v, sz(1), sz(2));
> ```
>
>
>
>
> **See also:** sparse.

— Function File: [err, y1, ...] = **common\_size** (x1, ...)  
> Determine if all input arguments are either scalar or of common
> size. If so, err is zero, and yi is a matrix of the
> common size with all entries equal to xi if this is a scalar or
> xi otherwise. If the inputs cannot be brought to a common size,
> errorcode is 1, and yi is xi. For example,
>
> ```
>           [errorcode, a, b] = common_size ([1 2; 3 4], 5)
>                => errorcode = 0
>                => a = [ 1, 2; 3, 4 ]
>                => b = [ 5, 5; 5, 5 ]
> ```
>
> This is useful for implementing functions where arguments can either
> be scalars or of common size.
